# Vortex Ratchet Effect in a NbC Strip With a Periodic Edge Indentation

**DOI:** 10.1002/smtd.202501430

**Published:** 2025-10-21

**Authors:** F. Porrati, A. O. Pokusinskyi, S. Barth, M. Huth, O. V. Dobrovolskiy

**Affiliations:** ^1^ Physikalisches Institut Goethe‐Universität Max‐von‐Laue‐Str. 1 60438 Frankfurt am Main Germany; ^2^ Cryogenic Quantum Electronics Institute for Electrical Measurement Science and Fundamental Electrical Engineering Technische Universität Braunschweig Hans‐Sommer‐Str. 66 38106 Braunschweig Germany; ^3^ Laboratory for Emerging Nanometrology (LENA) Technische Universität Braunschweig Langer Kamp 6A/B 38106 Braunschweig Germany

**Keywords:** focused ion beam induced deposition, nanofabrication, ratchet effect, superconductivity, TDGL simulation, vortex matter

## Abstract

The motion of magnetic flux quanta in superconductors leads to dissipation, making its control crucial for fluxonic devices. Recently, interest has grown in the ratchet (diode) effect, which enables non‐reciprocal, dissipationless currents and superconducting rectifiers, with potential applications in energy‐efficient computing, memory, and switching systems. However, most approaches to superconducting ratchet systems involve patterning thin films across their entire area, and few studies have examined symmetry breaking from disparities in thin‐film edge barriers. Here, non‐reciprocal current flow and vortex dynamics are demonstrated in a superconducting NbC strip with periodic edge indentations, which induce current crowding and edge barrier suppression, facilitating vortex entry. Upon reversing the current polarity at a magnetic field of 16 mT, a maximum ratchet efficiency of ≈35% is observed based on critical current differences, and ≈60% based on maximal voltage differences preceding the flux‐flow instability. Numerical simulations using the time‐dependent Ginzburg–Landau equation support the findings and reveal the formation of “flux pockets”‐regions where vortices become trapped between indentations—as well as diverse vortex configurations, including chains, jets, and rivers.

## Graphical abstract

The image shows the nanoprinting of a superconducting nanostructure using focused ion beam induced deposition (top left), overlaid with numerically simulated patterns of current and magnetic flux quanta (bottom). An edge indentation breaks spatial symmetry, modifying the current density distribution and inducing nonreciprocal magnetic flux dynamics the vortex diode (ratchet) effect

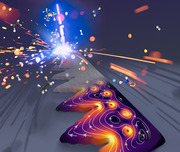

## 1 Introduction

The lack of spatial inversion symmetry gives rise to a range of phenomena in condensed matter physics, including ferroelectricity, high‐harmonic generation, and magnetochiral anisotropy.^[^ ^1^, ^2^ ^]^ In superconductors, symmetry breaking leads to nonreciprocal charge transport,^[^ ^3^ ^]^ as well as to rectification effects such as the diode or ratchet effect.^[^ ^4^, ^5^, ^6^, ^7^, ^8^, ^9^, ^10^, ^11^ ^]^ The ratchet effect describes rectified net transport of magnetic flux quanta (vortices) under the influence of zero time‐average forces, arising from spatial asymmetries in the pinning landscape.^[^ ^12^ ^]^ This phenomenon is of significant relevance to superconducting devices that rely on the controlled manipulation of vortex dynamics. In particular, rectification effects could be harnessed for novel data processing concepts and energy‐efficient superconducting computing.^[^ ^3^, ^12^, ^13^ ^]^ More broadly, interest in ratchets spans multiple disciplines, as rectified net transport has been experimentally observed in a variety of systems, including Josephson junctions,^[^ ^14^ ^]^ superconducting quantum interference devices,^[^ ^15^ ^]^ cold atoms,^[^ ^16^ ^]^ and the dynamics of domain walls,^[^ ^17^ ^]^ skyrmions,^[^ ^18^ ^]^ and colloidal particles.^[^ ^19^ ^]^ Ratchets have also been studied across thermodynamics,^[^ ^20^ ^]^ mechanics,^[^ ^21^ ^]^ and biology.^[^ ^22^ ^]^

In the presence of an asymmetric pinning potential, the motion of magnetic flux quanta (Abrikosov vortices or fluxons) and the resulting resistive response in superconducting films depend on the polarity of the applied current.^[^ ^12^ ^]^ Asymmetric pinning potential landscapes can be engineered through various approaches, such as film patterning with conformal arrays of holes,^[^ ^9^ ^]^ magnetic pinning centers,^[^ ^5^ ^]^ and washboard pinning structures.^[^ ^23^ ^]^

Most theoretical approaches to superconducting ratchet systems assume an infinite medium without boundaries or employ periodic boundary conditions. However, in real systems, vortex penetration into the superconductor is hindered by various surface and edge barriers,^[^ ^24^ ^]^ among which the Bean‐Livingston^[^ ^25^ ^]^ and the geometrical^[^ ^26^ ^]^ barrier are most essential. For superconducting thin strips with thickness *d* much smaller than the magnetic penetration depth, *d* ≪ λ, the vortex–vortex interaction length scale is governed by the effective (Pearl) penetration depth λ_eff_ = λ^2^/*d* ^[^ ^27^, ^28^ ^]^ and the effects of the geometrical barrier become negligible.^[^ ^29^ ^]^

Previous studies discussed the emergence of dc voltages generated by ac‐driven vortices in superconducting constrictions with engineered corrugations^[^ ^30^ ^]^ and unintended current inhomogeneity,^[^ ^31^ ^]^ and in Dayem nanobridges.^[^ ^32^ ^]^ The major effects arising from a pronounced edge defect are the bending of current streamlines^[^ ^33^ ^]^ due to the current‐crowding effect,^[^ ^34^ ^]^ and the reduction of the strip's cross‐section, which leads to a local enhancement of the transport current density.^[^ ^35^ ^]^ Together, these effects result in a local reduction of the edge barrier, facilitating vortex entry into the superconductor.^[^ ^36^ ^]^ Vortices penetrating the superconductor form a jet that is initially narrow near the defect and gradually broadens due to vortex–vortex repulsion as they move toward the opposite edge of the strip.^[^ ^37^, ^38^ ^]^ As the transport current increases, the edge defects turn into the nucleation points for vortex rivers whose emergence precedes the flux‐flow instability (FFI).^[^ ^39^ ^]^ The FFI arises when the electric field from vortex motion elevates quasiparticle energy above the energy potential barrier around the vortex core, allowing unpaired electrons to escape from it. This causes the vortex core to shrink, reducing viscous drag and triggering an abrupt vortex acceleration that quenches the low‐resistive state. Herewith, the shape of the defects affects the vortex dynamics^[^ ^37^ ^]^ and constrictions with multiple defects should allow for coexistence and interplay between various configurations of vortices, including vortex chains, vortex jets, and vortex rivers.

Here, we study a vortex ratchet effect in a NbC strip featuring periodic indentations (notches) along one of its edges. The NbC strip is fabricated by the direct‐write technique of focused ion beam induced deposition (FIBID). At an external magnetic field of 16 mT, the studied system exhibits a maximal ratchet efficiency of ≈35% for the critical current and ≈60% for the voltage just before the FFI onset. The observed non‐reciprocity in magnetic flux transport is corroborated by numerical modeling relying upon the time‐dependent Ginzburg‐Landau (TDGL) equation. The TDGL simulations suggest the pronounced ratchet effect to be attributed to a crossover from global to local dynamics upon current polarity reversal. Namely, in the former regime, vortices nucleate at many places at the entire straight edge of the strip, while in the latter case, vortices nucleate at the tips of the notches. Furthermore, the simulations suggest the formation of “flux pockets”—regions between the notches—that trap vortices for one current polarity due to locally enhanced barriers preventing their escape from the superconducting constriction. Overall, our findings advance the understanding of nonreciprocal vortex dynamics, which are crucial for developing future superconducting devices that operate in polarity‐dependent, dissipation‐free regimes.

## 2 Experimental Results

### 2.1 System Under Consideration

**Figure 1:**
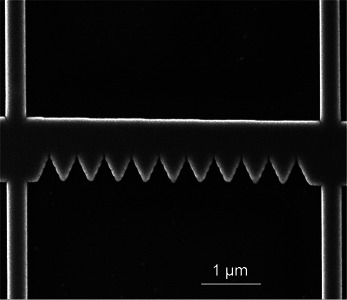
SEM image of the investigated NbC strip. The notch‐to‐notch distance is 475 nm.

The vortex dynamics is studied for a 5 µm‐long and 1 µm‐wide NbC strip with a thickness of 90 nm. One edge of the strip features 10 triangular‐shaped notches spaced 475 nm apart, see **Figure**  **1**. The strip was fabricated by FIBID using a Ga^+^ liquid source and Nb(NMe_2_)_3_(N‐t‐Bu) as precursor gas,^[^ ^40^ ^]^ see Methods for details. Electrical transport measurements were performed in the temperature range of 1.8–300 K using a standard four‐probe geometry in the current‐driven regime.

**Figure 2:**
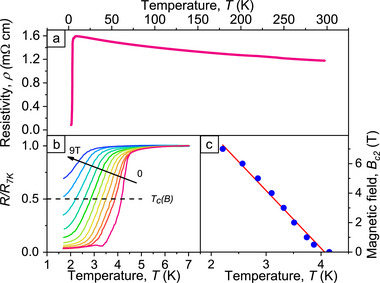
Superconducting properties of the NbC strip. a) Temperature dependence of the resistivity. b) Superconducting transition as a function of the magnetic field applied perpendicularly to the strip surface. In zero field, a residual resistance of 4% is measured at 2 K. c) Symbols: Experimental temperature dependence of the upper critical field. Line: Fit by the expression *B*_*c*2_(*T*) = *B*_*c*2_(0) − *T* · *dB*_*c*2_/*dT* with *B*_*c*2_(0) = 15.9 T and *dB*_*c*2_/*dT* = −3.91 T/K.

**Figure** **2**a shows the temperature dependence of the resistivity ρ(*T*) of the NbC strip. Upon cooling, the ρ(*T*) curve exhibits a transition from a weak localization behavior, typical for dirty metals, to a superconducting regime below the transition temperature *T*_*c*_ = 4.15 K, as deduced by using a 50% resistance drop criterion. The width of the superconducting transition is Δ*T* = 0.94 K, as determined by the temperature interval at 90% and 10% of the resistance at 7 K. The resistivity at 7 K is ρ_7K_ = 1.58 mΩcm.

The application of a magnetic field shifts *T*_*c*_ toward lower values, see Figure  2b. From this plot, the values of the upper critical field *B*_*c*2_(*T*) can be determined, see Figure  2c. Namely, the extrapolation toward zero temperature yields *B*_*c*2_(0) = 15.9 T, the slope *dB*_*c*2_/*dT* = −3.91 T/K, and the electron diffusion coefficient $D=-4k_{B}/\pi e\left(dB_{c2}/dT\right){|}_{T_{c}}\approx 0.28$ cm^2^s^−1^. The coherence length and the penetration depth at zero temperature are estimated^[^ ^41^ ^]^ as ξ(0)=$\sqrt{\hbar D/\Delta (0)}=8.6$ nm and $\lambda \left(0\right)=1.05\cdot 10^{-3}\sqrt{\rho_{7K}/T_{c}}=2048$ nm, where Δ(0) = 1.76*k*_*B*_ *T*_*c*_ is the BCS superconducting energy gap. The Pearl length Λ = λ^2^(0)/*d* ≈ 46 µm. Therefore, the strip is thin since *d* ≪ λ(0) and it is narrow, ξ(0) ≪ *w* ≪ Λ(0), where *w* is the strip width. This regime differs from the previously studied wide Al films with Λ(0) < ξ(0) ≪ *w* in ref. [30].

### 2.2 Current–Voltage Curves

**Figure 3:**
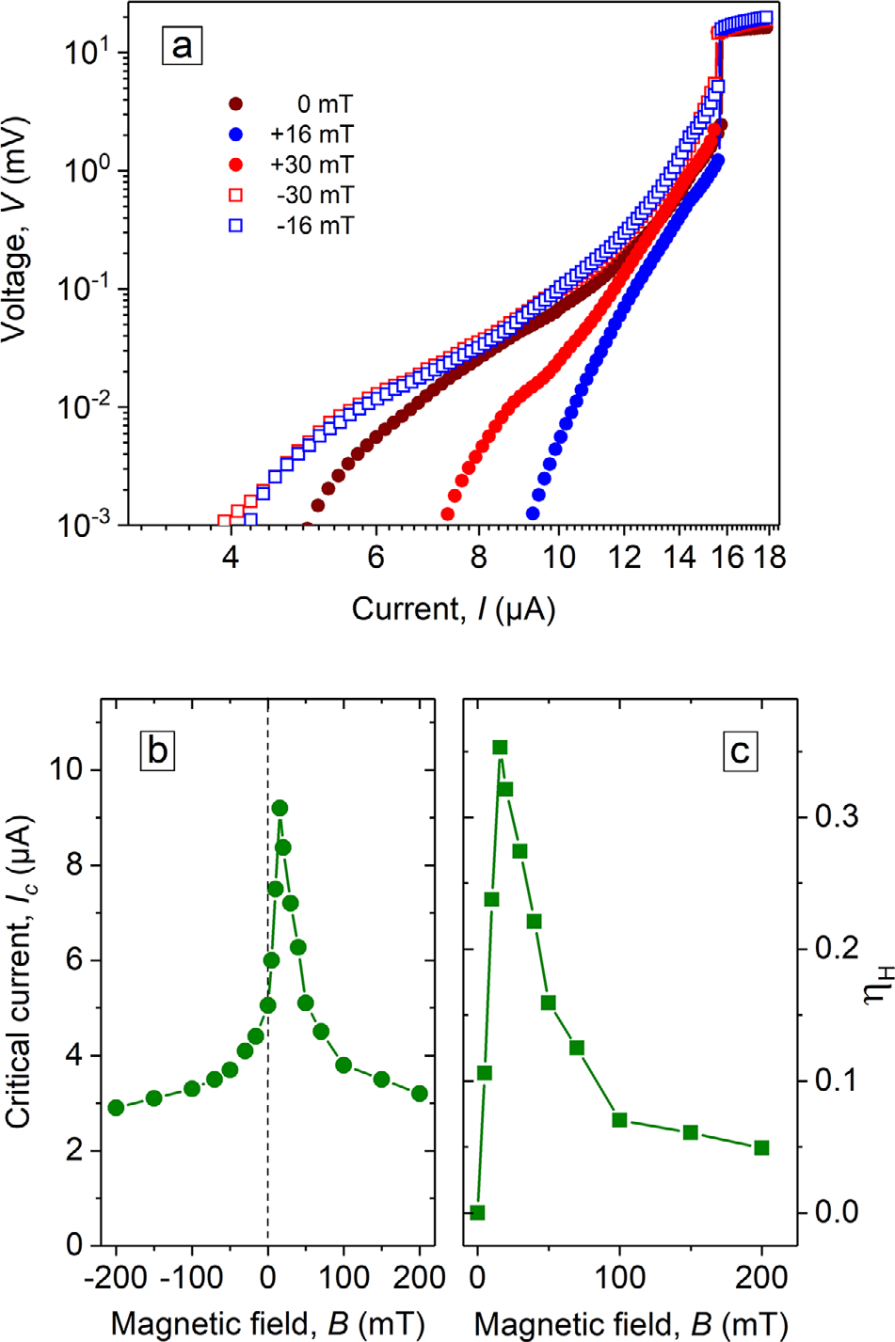
a) *I*–*V* curves of the NbC strip for a series of magnetic fields at 1.5 K. b) Dependence of the critical current *I*_*c*_ on the magnetic field *B*. c) Dependence of the vortex ratchet efficiency η_*H*_ on the magnetic field *B*.

**Figure** **3**a displays the *I*–*V* curves of the NbC strip for a series of magnetic fields at 1.5 K. It can be seen that the application of a magnetic field of +16 mT leads to a reduction of dissipation, and even at +30 mT the dissipation is less than at *B* = 0 up to a current of ≈12 µA. Conversely, applying a magnetic field of negative polarity leads to a gradual increase in dissipation, particularly at lower currents.

From the *I*–*V* curves, the critical current *I*_*c*_ was determined using a 1 µV criterion, after subtraction of the residual resistance. In general, the critical current of superconducting strips without edge defects is expected to be symmetric with respect to the reversal of the applied magnetic field direction.^[^ ^41^ ^]^ The presence of defects, however, diminishes the critical current^[^ ^29^, ^42^ ^]^ and, if only one edge contains defects, an asymmetric behavior is expected.^[^ ^38^, ^41^, ^43^ ^]^ Thus, the presence of the indents on one side of the NbC strip results in an asymmetry of the *I*_*c*_(*B*) upon field polarity reversal, as shown in Figure  3b.

The differences in critical values upon current polarity reversal can be quantified using the ratchet (rectification) efficiency parameter η_*H*_ = (*I*_*c*_(*H* ^+^) − *I*_*c*_(*H* ^−^))/(*I*_*c*_(*H* ^+^) + *I*_*c*_(*H* ^−^)). The calculated parameter η_*H*_ attains a maximum value of 35% at *B* = 16 mT, see Figure  3c. Note, in a previous study,^[^ ^41^ ^]^ we showed that no ratchet effect occurs in a strip with straight edges, whereas the introduction of a single edge defect leads to the emergence of asymmetry in the critical current under magnetic‐field reversal.

### 2.3 Fast Vortex Dynamics

**Figure 4:**
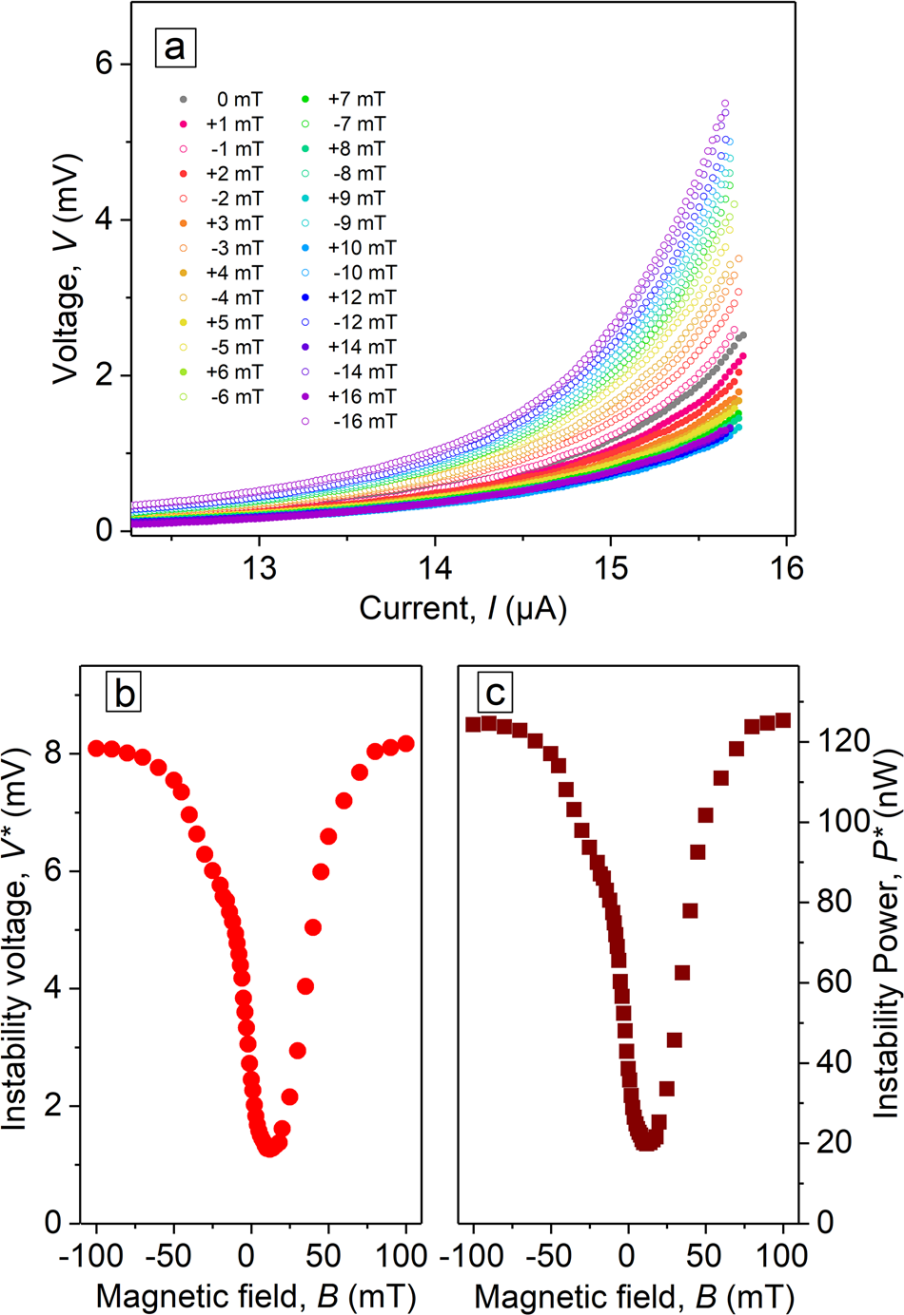
a) *I*–*V* curves in the high‐current regime. From the last points, the instability current I* and v* are deduced. b) Instability voltage V* and c) instability power P* in the range as a function of the magnetic field.

**Figure** **4**a displays the *I*–*V* curves in the high‐current regime for a series of magnetic fields of both polarities. All *I*–*V* curves exhibit a nonlinear upturn (regime of nonlinear conductivity) preceding an abrupt jump to a highly resistive state. From the last point in the *I*–*V* curves in Figure  4a, we deduce the instability current I* associated with the instability voltage V*.

In the absence of magnetic field V* = 2.4 mV. With increasing *B* the voltage V* is decreasing, reaching a minimum of 1.2 mV at *B* = 12 mT, see Figure  4b. For higher *B*, V* monotonically increases up to 8.1 mV at *B* = 100 mT. For negative values of *B*, the increase of V* is monotonic up to the same value. The instability power P* in Figure  4c follows the same behavior in the range of values 18 nW≪P* ≪ 125 nW.

**Figure 5:**
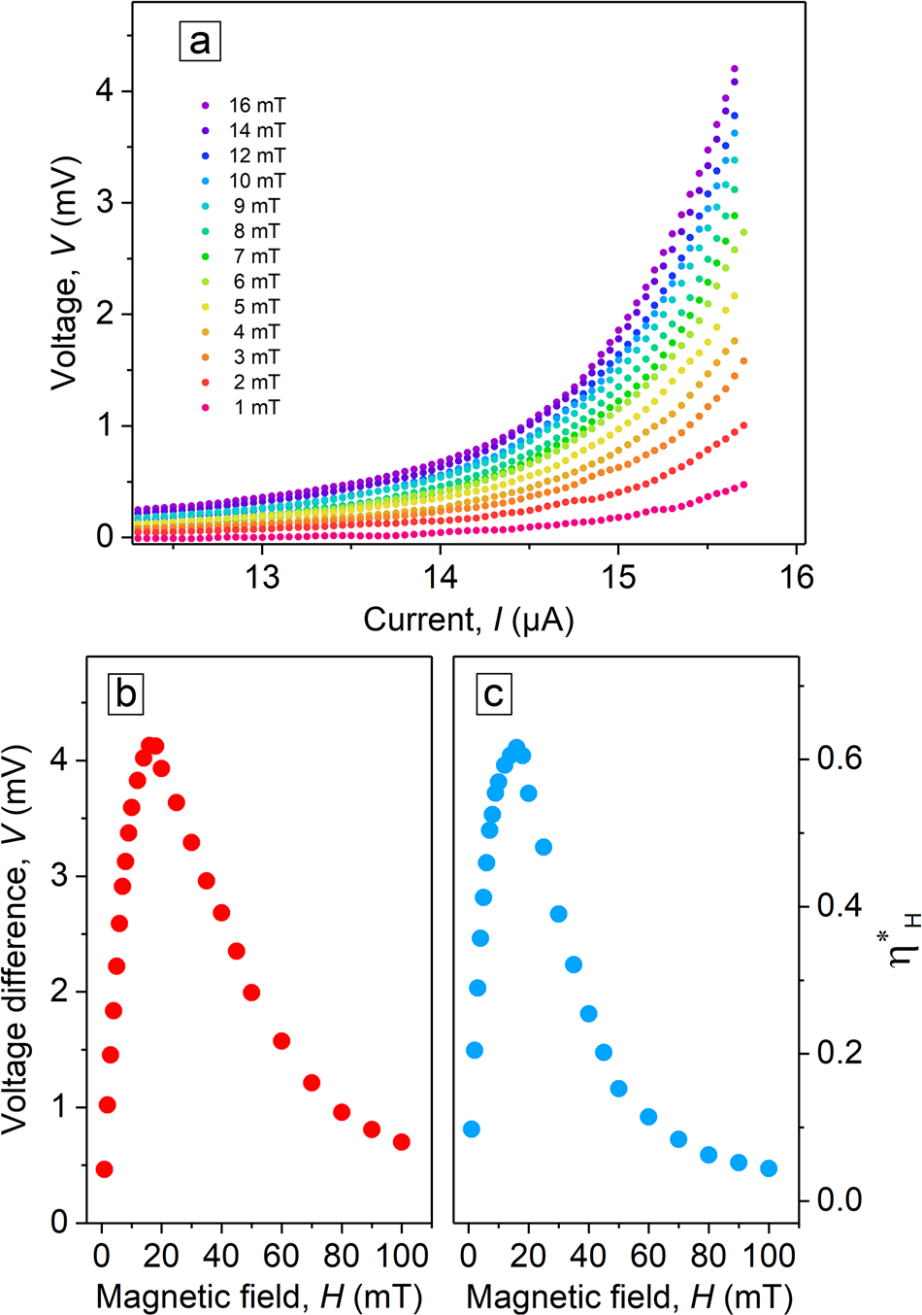
a) Evaluation of the voltage response upon *B* polarity reversal. For each value of *B*, the *I*–*V* curves are obtained by the difference of the curves plotted in Figure  4. b) Dependence of the voltage difference ΔV* = V*(− *B*) − V*(*B*) at the instability point on the magnetic field *B*. c) Dependence of the ratchet efficiency parameter $\eta_{H}^{*}$ on *B*.

To quantify the effect of the sign change of *B*, we subtract the *I*–*V* curves obtained for negative and positive *B* values, see **Figure**  **5**a. For each value of *B*, the largest difference of Δ*V* = *V*(*H* ^−^) − *V*(*H* ^+^) is obtained at the instability point, ΔV*. Figure 5b displays the dependence of ΔV* on the applied magnetic field *B*. The peak voltage difference of 4.1 mV at 16 mT aligns with the magnetic field value where the vortex ratchet efficiency is maximized.

In analogy with the vortex ratchet efficiency parameter η_*H*_ defined for the critical current *I*_*c*_, we introduce the parameter $\eta_{H}^{*}=\left(V^{*}\left(H^{-}\right)-V^{*}\left(H^{+}\right)\right)/\left(V^{*}\left(H^{-}\right)+V^{*}\left(H^{+}\right)\right)$ for the instability voltage V*. Figure 5c depicts the dependence of $\eta_{H}^{*}$ on the magnetic field *B*. Namely, $\eta_{H}^{*}\left(B\right)$ first increases up to a maximum value of 61% at *B* = 16 mT. For larger *B* > 16 mT, $\eta_{H}^{*}\left(B\right)$ exhibits a monotonic decrease. Note that both η_*H*_ and $\eta_{H}^{*}$ have a maximum at *B* = 16 mT.

In principle, by using the standard relation v* = V*/*BL*, where *L* is the distance between the voltage leads, it is possible to deduce the instability velocity v* from the last voltage point at the foot of the FFI jump. However, for the studied system featuring an incomplete superconducting transition, with a residual resistance of 4% at 2 K in zero field, such an analysis is not expected to yield reliable quantitative results at low magnetic field values. At the same time, our analysis has yielded v* ≈ 8 km s^−1^ at magnetic fields of the order of 100 mT, in line with those previously obtained for a NbC strip without indents.^[^ ^41^ ^]^

## 3 Modeling Results and Discussion

**Figure 6:**
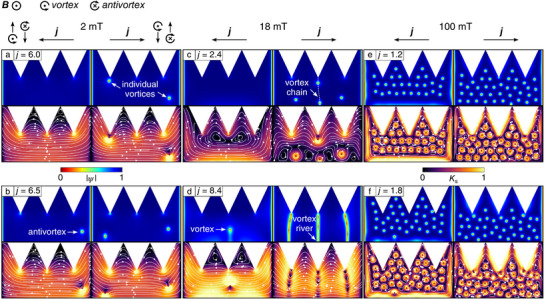
a–f) Representative snapshots of the spatial distribution of the absolute value of the superconducting order parameter |ψ| and the sheet current density *K*_*s*_ (in units of 0.01*K*_0_) obtained from the TDGL modeling. Simulations are done for a series of dc current densities *j* (in units of 0.01*j*_0_) and magnetic field values *B*. Each panel displays a 2D contour plot for |ψ| and *K*_*s*_ for negative (left) and positive (right) current polarities, corresponding to vortex motion from bottom to top and from top to bottom, respectively.

To explain the maximum in the vortex ratchet effect at 16 mT and the corresponding vortex configurations, numerical simulations of vortex dynamics were carried out based on the TDGL equation. The TDGL equation describes the spatiotemporal evolution of the superconducting order parameter ψ(**r**, *t*). Details on the TDGL equation, boundary conditions, and simulation parameters are given in the Experimental Section. The TDGL modeling was carried out for magnetic fields ranging from 0 to 100 mT, and we did not take into account possible overheating effects associated with the escape of non‐equilibrium phonons into the substrate as in ref. [41]. This simplification is justified since we are mainly interested in the initial section of the *I*–*V* curves where the overheating effects are negligible.^[^ ^37^ ^]^ The *I*–*V* curves were calculated for both transport current polarities, in order to determine the critical current densities, $j_{c}^{+}$ and $j_{c}^{-}$. The obtained snapshots of the superconducting order parameter and the *I*–*V* curves are presented in **Figures**  **6** and **8**, respectively.

**Figure 7:**
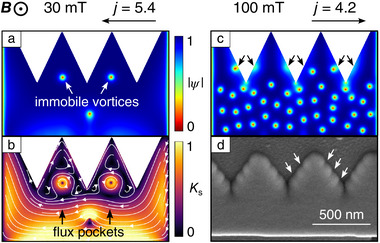
Contour plots of |ψ| (a) and *K*_*s*_ (b) (same units as in Figure  6) for negative current polarity at 30 mT. c) Contour plot for |ψ| for positive current polarity at 100 mT. Black arrows indicate additional locations of the vortex nucleation. d) Enlarged part of the SEM image of the NbC strip. Smaller defects along the edges of one of the indents are indicated by white arrows.

**Figure 8:**
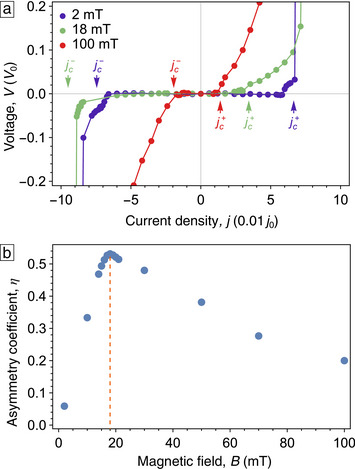
a) Calculated *I*–*V* curves of the NbC strip with a periodic edge indentation at magnetic fields of 2, 18, and 100 mT. Critical current densities are indicated for the negative ($j_{c}^{-}$) and positive ($j_{c}^{+}$) current polarities. b) Calculated dependence of the ratchet efficiency parameter η on the applied magnetic field *B*.

At small magnetic fields, see the data for 2 mT in Figures  6a,b and 8a, vortices only penetrate via the edge with the indents and there is no vortex penetration through the straight edge. Note that positive current polarity induces vortex nucleation at locations with the highest current density (i.e., at the tips of the notches), while negative current polarity results in antivortex nucleation at the same sites. Accordingly, the critical current $|j_{c}^{-}|$ is slightly higher than $|j_{c}^{+}|$.

As the magnetic field *B* increases, antivortex penetration into the sample becomes suppressed. When *B* exceeds 18 mT, for negative current polarity, the sheet current density increases at the straight edge, the edge barrier is suppressed, and the vortices begin to penetrate via the straight edge as well, see Figure  6c,d. In this regime, $|j_{c}^{-}|$ is significantly larger than $|j_{c}^{+}|$, but this difference begins to decrease with a further increase of *B*.

At moderately strong magnetic fields (≈30 mT), and as long as the current does not reach the critical value, vortices begin to accumulate near the edge of the sample. The edge indentations significantly alter the local current distribution. When combined with the screening currents induced by the external magnetic field, this results in a pronounced reduction in sheet current density near and within the “teeth” regions under negative current polarity, see Figure  6c,d. This effect leads to the formation of regions where the current density is insufficient to push vortices further toward the indented edge. As a result, “flux pockets” emerge as areas with immobile flux quanta, see also **Figure**  **7**a,b.

At high *B* values, under positive current polarity, vortices penetrate the sample not only through the tips of the notches but also at other sites, see Figures  6f and 7c. Indeed, a closer inspection reveals an increase in the sheet current density distribution within the “teeth” between the notches. We believe that the penetration of vortices at certain places along the teeth edges is possible in the experiment, especially for higher flux densities, see Figure 7c. Similarly, the enlarged SEM image in Figure 7d reveals some smaller edge defects, whose occurrence is attributed to the rastering process during FIBID. In this regime, the difference between $|j_{c}^{-}|$ and $|j_{c}^{+}|$ decreases further.

**Figure 9:**
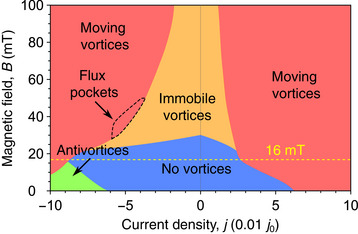
Phase diagram obtained from the TDGL simulations. The field of ≈16 mT, indicated by the yellow dashed line, corresponds to both the maximum simulated ratchet efficiency and the field value at which antivortices stop entering the sample.

The *I*‐*V* curves corresponding to the *B* values in Figure  6 are shown in Figure  3a. The ratchet efficiency parameter η is deduced from the *I*–*V* curves in Figure  6 by the formula

(1) $$\eta =\left|\frac{|j_{c}^{+}\left|-\right|j_{c}^{-}|}{|j_{c}^{+}\left|+\right|j_{c}^{-}|}\right|$$

where $j_{c}^{+}$ is the critical density for the positive current polarity and $j_{c}^{-}$ for the negative. The parameter η calculated by Equation (1) is presented in Figure  8b as a function of the magnetic field. At small magnetic fields, η first increases, then it reaches a maximum value of about 53% at 18 mT. This numerical value of η is larger than the experimental one of 35% at 16 mT in Figure  3c. This difference can be explained as follows. Our model idealizes the sample by assuming a perfectly smooth, straight edge. In contrast, the sample's nominally flat edge, fabricated by FIBID, exhibits small imperfections and roughness, see Figure 7d. Namely, during the FIBID process, the ion beam moves point‐by‐point along a square lattice with a constant pitch of 30 nm. Because the indent's width is not a multiple of 30 nm, its triangular shape means that the edges of the indent can miss some of the raster points. These missed points are the primary source of the small edge defects, which, in turn, reduce the difference in the edge barrier between the indented and non‐indented sides of the sample. In contrast, the absence of such imperfections in the simulations leads to a stronger difference between the edge barriers. As a result, the numerically obtained ratchet efficiency is overestimated compared to the experimental one. With a further increase of *B*, the ratchet efficiency gradually decreases, see Figure  8b. In this way, the maximal ratchet efficiency in the simulations is in line with the experimental observations of the maxima in η_*H*_(*B*) and $\eta_{H}^{*}\left(B\right)$, as reported in Figures  3c and 5c. The overall trends of increasing η_*H*_(*B*) at small fields and decreasing η_*H*_(*B*) at larger fields are also reproduced in simulations. These trends are further illustrated in a phase diagram in **Figure**  **9**, which shows the evolution of different vortex regimes as a function of the magnetic field and the transport current.

Figure 9 summarizes the vortex regimes revealed in the TDGL modeling. The area, labeled “No vortices”, corresponds to a vortex‐free regime in Figure  6a,c for the negative current, and this regime transits to a regime of immobile vortices with an increase of the magnetic field. In this regime, vortices penetrate the sample but remain pinned by the surface barrier, see Figure  6e for the negative current. The areas labeled “Moving vortices” correspond to the regimes where vortices co‐aligned with the applied magnetic field enter the sample and annihilate at the opposite edge, see Figure  6a–d for the positive current and Figure  6e,f. Finally, the small area labeled “Antivortices”, corresponds to the regime where antivortices start to enter the sample from the indented edges at negative currents, see Figure  6b for the negative current.

The vortex‐free regime in Figure  9 is determined by edge‐barrier effects that inhibit vortex penetration. At zero magnetic field, this vortex‐free regime remains symmetric under current‐polarity reversal. A positive current drives vortices to enter from the indented side of the sample, whereas a negative current drives antivortices to enter from the same indented side, see Figure  6b for the negative current. The application of a magnetic field breaks the symmetry of the vortex‐free regime, extending it to higher negative currents for fields up to 16 mT. In this case, antivortices can only penetrate from the indented side under negative current, while the magnetic field enhances the surface barrier against their entry. At higher fields, where vortices are always present in the sample, a “Flux pockets” region emerges between the “Immobile vortices” and “Moving vortices” regimes at negative currents. This region originates from current crowding, which creates a zone of minimal current density within the sample. Vortices trapped in this zone, see Figure  7b, require currents several times larger to be depinned and set into motion. In principle, for materials with a smaller magnetic penetration depth (e.g., Pb),^[^ ^33^ ^]^ the presence of immobile vortices could be verified using scanning SQUID‐on‐tip microscopy. For dirty‐limit superconductors with a large magnetic penetration depth, such as the NbC studied here, detection of trapped vortices via the SQUID‐on‐tip technique is more challenging, but could, in principle, be feasible^[^ ^45^ ^]^ if the predicted effect occurs in wider strips.

We emphasize that our TDGL simulations are restricted to the regimes of weak and moderately strong currents. The FFI regime, in which the experiment reveals a higher ratchet efficiency of 61%, see Figure  5c, lies beyond the applicability of our simulations. This limitation arises because, in the FFI regime, local heating plays a significant role. Accurately capturing this behavior would require solving the heat‐balance equation alongside the TDGL equations.^[^ ^41^ ^]^ Since our current computational framework does not include thermal effects, simulations in the instability regime fall beyond the scope of this study.

Finally, we would like to comment on the maximum of the ratchet efficiency occurring at *B* = 16 mT. The vortex arrangement for positive current polarity in panel (c) of Figure  6 suggests a possible commensurability between the vortex spacing and the periodicity of the indentation. Specifically, while vortex penetration is impeded by the strong barrier of the straight edge under negative current polarity, for positive polarity the vortices form a pattern reminiscent of a compressed hexagonal lattice. For an undistorted hexagonal vortex lattice in a wide superconducting film where ξ(0) ≪ Λ < *w*, the expected vortex lattice parameter is *a* = (4/3)^1/4^(Φ_0_/*B*)^1/2^ = 475 nm at *B* = 10.6 mT. However, in narrow constrictions with ξ(0) ≪ *w* ≪ Λ, as investigated in this work, edge‐barrier effects significantly impede vortex entry. As a result, the characteristic vortex spacing is achieved only at much higher magnetic fields than those predicted by the flux quantization estimate *n*Φ_0_ = *BS* where *n* = 1, 2, …, Φ_0_ is the magnetic flux quantum, and *S* is the sample surface area.^[^ ^41^ ^]^

As a final point of our analysis, we note that varying the material parameters in the TDGL simulations reveals a shift of the η maximum toward higher magnetic fields for materials with smaller ξ, accompanied by an overall increase in η.

## 4 Conclusion

Summarizing the results of our studies of the ratchet effect in the NbC strip with a periodic edge indentation, our main findings can be outlined as follows. At small magnetic fields, the vortices and antivortices are generated by the self‐field of the transport current and penetrate through the edge with the indentation. An increase of the magnetic field decreases the surface barrier for vortices and increases it for antivortices. This leads to an increase of the ratchet efficiency which attains a maximum at 16 mT. At magnetic fields exceeding 16 mT, the edge barrier for vortices is decreasing for the straight edge as well. Due to a non‐uniform distribution of the transport current within the “teeth” regions under negative current polarity, there appear regions where the current density is insufficient to push vortices further toward the indented edge, leading to the appearance of “flux pockets” as areas with immobile flux quanta. With a further increase in the number of vortices, the vortices eventually begin to penetrate through the smaller defects at the indent edges, and the ratchet effect gradually vanishes with a further increase of the magnetic field.

## 5 Materials and Methods

### Fabrication and Characterization

The sample was fabricated by Ga^+^ FIBID in a dual‐beam scanning electron microscope (FIB/SEM: FEI, Nova NanoLab 600) equipped with a Schottky electron emitter. In FIBID, the adsorbed molecules of a precursor gas injected in a FIB/SEM microscope dissociate under the impact of the ion beam, thus forming the sample during the rastering process. The precursor, Nb(NMe_2_)_3_(N‐t‐Bu), was introduced into the microscope chamber through a standard gas‐injection system with a capillary of 0.5 mm inner diameter. The angle between the capillary and the ion beam was 35 degrees. The capillary‐substrate distance was 100 µm. The basis pressure in the microscope was 5 × 10^−7^ mbar, which increased to about 2 × 10^−6^ mbar during the deposition. The temperature of the precursor gas was 35°C. The ion beam parameters used during the deposition were 30 kV acceleration voltage, 10 pA beam current, 30 nm pitch, and 200 ns dwell time. The geometry of the sample was defined by shaping a polygonal structure using the standard FEI software. During the deposition, the ion beam was rastered over the structure in a serpentine mode. Following deposition, the NbC strip was capped, using focused electron beam induced deposition (FEBID), with a highly resistive layer from the same precursor. FEBID was done with 5 keV acceleration voltage, 1.6 nA beam current, 20 nm pitch, and 1 µs dwell time. The sample was fabricated on a Si/SiO_2_ substrate with ca. 100 nm thick Au/Cr electrodes prepared by standard UV‐lithography.

Electrical transport measurements were carried out in a variable‐temperature insert in a ^4^He cryostat equipped with a 11 T superconducting solenoid employing a Keithley Sourcemeter 2635B and an Agilent 34420A nanovoltmeter. The magnetic field was oriented perpendicular to the strip surface.

### TDGL Simulations

In the 2D geometry, the generalized TDGL^[^ ^45^, ^46^ ^]^ reads

(2) $$\begin{array}{cc} \frac{u}{\sqrt{1+\gamma^{2}{\left|\psi \right|}^{2}}} & \left(\frac{\partial }{\partial t}+i\varphi +\frac{\gamma^{2}}{2}\frac{{\partial |\psi |}^{2}}{\partial t}\right)\psi \\ & ={\left(\nabla -iA\right)}^{2}{\psi +(1-|\psi |}^{2})\psi \end{array}$$

where *u* = π^4^/14ζ(3) ≈ 5.79 is the ratio of the relaxation times for the amplitude and phase of the order parameter in the dirty superconducting limit with ζ(*x*) being the Riemann zeta function, γ = 2τ_*E*_Δ_0_/ℏ is a parameter where τ_*E*_ is the inelastic electron‐phonon scattering time and Δ_0_ the zero‐field superconducting gap, φ(**r**, *t*) is the electric scalar potential, and **A**(**r**) is the vector potential.

The total current density is given by the sum of the supercurrent density **j**_*s*_ = Im[ψ*(∇ − *i* **A**)ψ] and the normal current density $j_{n}=-\nabla \varphi -\frac{\partial A}{\partial t}$. The electric potential φ(**r**, *t*) is described by the Poisson equation

(3) ∇2φ=∇·Im[ψ*(∇−iA)ψ]−∇·∂A∂t

which arises from the condition of continuity of the total current density ∇ · (**j**_*s*_ + **j**_*n*_) = 0.

For the edges where vortices enter and exit the strip, the boundary conditions for superconductor–vacuum interfaces read

(4) n·(∇−iA)ψ=0,n·∇φ=0

where **n** is a unit vector normal to the interface.

For the superconductor–normal metal interfaces, which are used to inject the transport current of density *j*, the boundary conditions read

(5) ψ=0,n·∇φ=j

The quantities in Eqs. (2) and (3) are expressed in the following dimensionless units. The superconducting order parameter |ψ| is in units of |ψ_0_|, the magnitude of the order parameter in the absence of applied fields or currents, 0 ⩽ |ψ|^2^ = *n*_*s*_ ⩽ 1, where *n*_*s*_ is the superfluid density normalized to its zero‐field value. The distance *r* is measured in units of the coherence length ξ, time *t* in units of τ_0_ = µ_0_σλ^2^, where σ is the normal conductivity and λ the London penetration depth. Magnetic field is measured in units of the upper critical field *B*_0_ = *B*_*c*2_ = Φ_0_/(2πξ^2^), where Φ_0_ = *h*/(2*e*) is the magnetic flux quantum. The vector potential is measured in units of *A*_0_ = ξ*B*_0_ = Φ_0_/2πξ and the current density in units of *j*_0_ = 4ξ*B*_*c*2_/(µ_0_λ^2^). The sheet current density is measured in units of *K*_0_ = *j*_0_ *d* = 4ξ*B*_*c*2_/(µ_0_Λ), where *d* is the film thickness and Λ = λ^2^/*d* is the effective (Pearl) penetration depth. The electric potential φ is measured in units of *V*_0_ = ξ*j*_0_/σ = 4ξ^2^ *B*_*c*2_/(µ_0_σλ^2^).

The TDGL simulations were performed for a 90 nm‐thick strip, but having only three indents with the teeth of the same size as in the experiment, see Figure  1: 1.6 µm length, 1 µm width, and 475 nm notch‐to‐notch distance. The material parameters used in the simulations are reported in **Table** **1**. The maximum mesh size was chosen as ξ/2. The TDGL simulations were carried out using the Python pyTDGL package.^[^ ^46^ ^]^

**Table 1:** Material parameters used in the simulations.

Parameter	Denotation	Value
Electron mean free path	*l*	2 nm
Inelastic scattering coefficient	γ	10
Temperature	*T*	4 K
Relative temperature	*t* = *T*/*T*_c_	0.96
Coherence length	$\xi =0.85\;\sqrt{\xi (0)l/(1-t)}$	19 nm
Penetration depth	$\lambda =0.615\;\lambda \left(0\right)\sqrt{\xi (0)/(l(1-t))}$	13.8 µm

## Conflict of Interest

The authors declare no conflict of interest.

## Data Availability

The data underlying this study are openly available in Mendeley Data at doi: 10.17632/zcmgjntfgn.

## References

[1] E. Goulielmakis , T. Brabec , Nat Photonics 2022, 16, 411.

[2] R. Wakatsuki , Y. Saito , S. Hoshino , Y. M. Itahashi , T. Ideue , M. Ezawa , Y. Iwasa , N. Nagaosa , Sci. Adv. 2017, 3, e1602390. 28439548 10.1126/sciadv.1602390 PMC5400453

[3] F. Qin , W. Shi , T. Ideue , M. Yoshida , A. Zak , R. Tenne , T. Kikitsu , D. Inoue , D. Hashizume , Y. Iwasa , Nat. Commun. 2017, 8, 14465. 28205518 10.1038/ncomms14465 PMC5316891

[4] C.‐S. Lee , B. Janko , I. Derenyi , A.‐L. Barabasi , Nature 1999, 400, 337.

[5] J. E. Villegas , S. Savel'ev , F. Nori , E. M. Gonzalez , J. V. Anguita , R. García , J. L. Vicent , Science 2003, 302, 1188. 14615532 10.1126/science.1090390

[6] C. C. de Souza Silva , J. Van de Vondel , M. Morelle , V. V. Moshchalkov , Nature 2006, 440, 651. 16572166 10.1038/nature04595

[7] D. Cole , S. Bending , S. Savel'ev , A. Grigorenko , T. Tamegai , F. Nori , Nat. Mater. 2006, 5, 305. 16532001 10.1038/nmat1608

[8] F. Ando , Y. Miyasaka , T. Li , J. Ishizuka , T. Arakawa , Y. Shiota , T. Moriyama , Y. Yanase , T. Ono , Nature 2020, 584, 373. 32814888 10.1038/s41586-020-2590-4

[9] Y.‐Y. Lyu , J. Jiang , Y.‐L. Wang , Z.‐L. Xiao , S. Dong , Q.‐H. Chen , M. V. Milošević , H. Wang , R. Divan , J. E. Pearson , P. Wu , F. M. Peeters , W.‐K. Kwok , Nat. Commun. 2021, 12, 2703. 33976211 10.1038/s41467-021-23077-0 PMC8113273

[10] Y. Hou , F. Nichele , H. Chi , A. Lodesani , Y. Wu , M. F. Ritter , D. Z. Haxell , M. Davydova , S. Ilić , O. Glezakou‐Elbert , A. Varambally , F. S. Bergeret , A. Kamra , L. Fu , P. A. Lee , J. S. Moodera , Phys. Rev. Lett. 2023, 131, 027001. 37505965 10.1103/PhysRevLett.131.027001

[11] P. J. Moll , Commun. Mater. 2025, 6, 73. 40236752 10.1038/s43246-025-00788-1 PMC11994448

[12] B. L. T. Plourde , IEEE Trans. Appl. Supercond. 2009, 19, 3698.

[13] P. Hänggi , F. Marchesoni , Rev. Mod. Phys. 2009, 81, 387.

[14] M. Knufinke , K. Ilin , M. Siegel , D. Koelle , R. Kleiner , E. Goldobin , Phys. Rev. E 2012, 85, 011122. 10.1103/PhysRevE.85.011122 22400527

[15] I. Zapata , R. Bartussek , F. Sols , P. Hänggi , Phys. Rev. Lett. 1996, 77, 2292. 10061907 10.1103/PhysRevLett.77.2292

[16] R. Gommers , S. Denisov , F. Renzoni , Phys. Rev. Lett. 2006, 96, 240604. 16907228 10.1103/PhysRevLett.96.240604

[17] A. Pérez‐Junquera , V. I. Marconi , A. B. Kolton , L. M. Álvarez‐Prado , Y. Souche , A. Alija , M. Vélez , J. V. Anguita , J. M. Alameda , J. I. Martín , J. M. R. Parrondo , Phys. Rev. Lett. 2008, 100, 037203. 18233032 10.1103/PhysRevLett.100.037203

[18] C. Reichhardt , C. J. O. Reichhardt , M. V. Milošević , Rev. Mod. Phys. 2022, 94, 035005.

[19] J. Rousselet , L. Salome , A. Ajdari , J. Prostt , Nature 1994, 370, 446. 8047163 10.1038/370446a0

[20] M. von Smoluchowski , Physikalische Zeitschrift 1912, 13, 1069.

[21] J. Bree , The effect of cyclic stress on engineering materials, Technical report, no DOI available.

[22] P. Reimann , Phys. Rep. 2002, 361, 57.

[23] O. Dobrovolskiy , E. Begun , V. Bevz , R. Sachser , M. Huth , Phys. Rev. Appl. 2020, 13, 024012.

[24] E. H. Brandt , Rep. Progr. Phys. 1995, 58, 1465.

[25] C. P. Bean , J. D. Livingston , Phys. Rev. Lett. 1964, 12, 14.

[26] E. Zeldov , A. I. Larkin , V. B. Geshkenbein , M. Konczykowski , D. Majer , B. Khaykovich , V. M. Vinokur , H. Shtrikman , Phys. Rev. Lett. 1994, 73, 1428. 10056790 10.1103/PhysRevLett.73.1428

[27] J. Pearl , Appl. Phys. Lett. 1964, 5, 65.

[28] V. G. Kogan , Phys. Rev. B 1994, 49, 15874. 10.1103/physrevb.49.15874 10010721

[29] G. P. Mikitik , Phys. Rev. B 2021, 104, 094526.

[30] D. Cerbu , V. N. Gladilin , J. Cuppens , J. Fritzsche , J. Tempere , J. T. Devreese , V. V. Moshchalkov , A. V. Silhanek , J. Van de Vondel , New J. Phys. 2013, 15, 063022.

[31] F. G. Aliev , A. P. Levanyuk , R. Villar , J. F. Sierra , V. V. Pryadun , A. Awad , V. V. Moshchalkov , New J. Phys. 2009, 11, 063033.

[32] D. Margineda , A. Crippa , E. Strambini , Y. Fukaya , M. T. Mercaldo , M. Cuoco , F. Giazotto , Commun. Phys. 2023, 6, 343.

[33] L. Embon , Y. Anahory , Ž. L. Jelić , E. O. Lachman , Y. Myasoedov , M. E. Huber , G. P. Mikitik , A. V. Silhanek , M. V. Milošević , A. Gurevich , E. Zeldov , Nat. Commun. 2017, 8, 85. 28729642 10.1038/s41467-017-00089-3 PMC5519736

[34] O.‐A. Adami , D. Cerbu , D. Cabosart , M. Motta , J. Cuppens , W. A. Ortiz , V. V. Moshchalkov , B. Hackens , R. Delamare , J. Van de Vondel , A. V. Silhanek , Appl. Phys. Lett. 2013, 102, 052603.

[35] J. R. Clem , K. K. Berggren , Phys. Rev. B 2011, 84, 174510.

[36] D. Y. Vodolazov , I. L. Maksimov , E. H. Brandt , Physica C: Superconductivity 2003, 384, 211.

[37] B. Budinská , B. Aichner , D. Y. Vodolazov , M. Y. Mikhailov , F. Porrati , M. Huth , A. Chumak , W. Lang , O. Dobrovolskiy , Phys. Rev. Appl. 2022, 17, 034072.

[38] A. I. Bezuglyj , V. A. Shklovskij , B. Budinská , B. Aichner , V. M. Bevz , M. Y. Mikhailov , D. Y. Vodolazov , W. Lang , O. V. Dobrovolskiy , Phys. Rev. B 2022, 105, 214507.

[39] O. V. Dobrovolskiy , Fast dynamics of vortices in superconductors , Elsevier, Amsterdam, ISBN 978‐0‐323‐90800‐9.00015‐9, 2024 pp. 735–754.

[40] F. Porrati , S. Barth , R. Sachser , O. V. Dobrovolskiy , A. Seybert , A. S. Frangakis , M. Huth , ACS Nano 2019, 13, 6287. 31046238 10.1021/acsnano.9b00059

[41] O. V. Dobrovolskiy , D. Y. Vodolazov , F. Porrati , R. Sachser , V. M. Bevz , M. Y. Mikhailov , A. V. Chumak , M. Huth , Nat. Commun. 2020, 11, 3291. 32620789 10.1038/s41467-020-16987-y PMC7335109

[42] A. Aladyshkin , A. S. Mel'nikov , I. A. Shereshevsky , I. D. Tokman , Physica C: Superconductivity 2001, 361, 67.

[43] D. Y. Vodolazov , F. M. Peeters , Phys. Rev. B 2005, 72, 172508.

[44] L. Ceccarelli , D. Vasyukov , M. Wyss , G. Romagnoli , N. Rossi , L. Moser , M. Poggio , Phys. Rev. B 2019, 100, 104504.

[45] L. Kramer , R. J. Watts‐Tobin , Phys. Rev. Lett. 1978, 40, 1041.

[46] L. Bishop‐Van Horn , Comput. Phys. Commun. 2023, 291, 108799.

